# Incidence of atrial high rate episodes after dual-chamber permanent pacemaker implantation and its clinical predictors

**DOI:** 10.1016/j.ihj.2022.11.013

**Published:** 2022-11-30

**Authors:** Jeffry Samuel, Akash Batta, Parag Barwad, Yash Paul Sharma, Prashant Panda, Navjyot Kaur, Y.S. Shrimanth, C.R. Pruthvi, Bharat Sambyal

**Affiliations:** a Department of Internal Medicine, Advanced Cardiac Centre, Post Graduate Institute of Medical Education & Research, Chandigarh, 160012, India; b Department of Cardiology, Dayanand Medical College and Hospital, Ludhiana, 141001, India; c Department of Cardiology, Advanced Cardiac Centre, Post Graduate Institute of Medical Education & Research, Chandigarh, 160012, India

**Keywords:** Atrial fibrillation, Dual-chamber pacemakers, Atrial high rate events, Apical pacing

## Abstract

Atrial high rate episodes (AHRE) confers increased morbidity and mortality amongst patients with permanent pacemaker implantation (PPI). The incidence of AHREs and it's clinical predictors in Indian patients without prior history of atrial fibrillation (AF) are not well understood. A total of 100 dual-chamber PPI patients, who had no prior history of AF, underwent pacemaker interrogation starting from a minimum of 1 month after implantation to detect any AHREs. The incidence of AHREs was 17% at a mean follow up 15.2 ± 7.5 months. Only right ventricular apical lead position was found to have an independent association with AHREs (OR: 3.50, 95% CI: 1.02–12.03; p = 0.04).

## 1 Introduction

Being the commonest cause of cardiac rhythm abnormality seen in clinical setting, atrial fibrillation (AF) is responsible for more physician visits compared to any other forms of cardiac arrhythmia and is associated with considerable morbidity and mortality.^1^^,^^2^ Since the implantation of first cardiac pacemaker in humans in 1958, there has been significant improvement in pacemaker design and technology resulting in establishment of near-normal cardiac hemodynamic post permanent pacemaker implantation (PPI). Current research favours the impanation of new generation dual-chamber permanent pacemaker which maintain atrioventricular synchrony. Besides improving cardiac hemodynamics, it also lowers the rate of AF, heart failure and stroke compared to single-chamber PPI.^3^^,^^4^

Despite the progress, there is evidence that even dual-chamber pacemakers are associated with reduction in ejection fraction, cardiac output and global longitudinal strains in the long run which suggests the negative impact of PPI on cardiac mechanics.^3^ This in turn predisposes to the development of increased left atrial pressure, volume and dysfunction which results in atrial high rate episodes (AHRE). Modern pacemakers can automatically record and store these AHRE according to programmable detection criteria. AHRE are defined as episodes of atrial tachyarrhythmia with rate >190 beats per minute detected by cardiac implantable electronic devices (CIED). These episodes of AHRE are believed to represent ill sustained AF which may be predictors of future sustained AF with attended deleterious effects on cardiovascular system causing excess mortality.^5^ Hence AHREs are a good surrogate of atrial tachyarrhythmia including both AF and atrial tachycardia (AT). Risk factors like advanced age, hypertension, left atrial enlargement, congestive heart failure, right ventricular apical pacing and sinus node dysfunction as indication for pacing have all been implicated as risk factors for the development of these AHREs, but conclusive evidence is lacking.^6^^,^^7^ Most of the prior studies done to find the incidence of AF after CIED implantation have included patients with preimplant AF and AF occurring within one month of implantation, thereby resulting in a much higher incidence of AF. New-onset AF after CIED implantation has significant long-term consequences in patients previously in sinus rhythm.^5^, ^6^, ^7^ The burden of new-onset AF and it's clinical predictors in Indian patients with dual-chamber CIED implantation from one month after implantation are not clearly known and our study aims to find this out.

## 2 Methods

### 2.1 Study population

This was a single centre, cross-sectional, prospective study conducted at a tertiary centre in North India enrolling 100 consecutive patients who had undergone dual-chamber PPI in the last 36 months. Patients having documented preimplantation AF at any time were excluded, All patients fulfilling the selection criteria underwent device interrogation at any time ≥1 month after the device implantation during the routine follow-up visit.

### 2.2 Inclusion criteria

1) Dual-chamber PPI within last 36 months
2) Absence of preimplantation AF
3) Interrogation available ≥1 month since device implantation
4) Willing for participation in the study

### 2.3 Exclusion criteria

1) PPI done >36 months prior to recruitment
2) Preimplantation AF documented at any time before implantation or AF occurring ≤1 month after implantation
3) Valvular lesions likely to cause AF

The common indication for PPI included sinus node dysfunction and atrioventricular (AV) block or advanced His-Purkinje disease. Any history of co-morbidities, previous coronary artery bypass surgery or percutaneous coronary intervention, use of medications like *β*-blockers, angiotensin converting enzyme (ACE) inhibitors, angiotensinII receptor blockers (ARB) and statins, were noted both at baseline and at follow-up. Baseline electrocardiogram before implantation to rule out AF and chest x-ray after implantation to note the ventricular lead position was done in all patients.

### 2.4 Follow up

During follow up, any time from one month after implantation, patients underwent electrocardiogram, detailed echocardiographic examination and device interrogation. Left atrial volume, left ventricular ejection fraction and any valvular lesions were noted in echocardiography. Pacemaker parameters like cumulative % of atrial and ventricular pacing, lead threshold, sensitivity and impedance and AHRE duration if any were recorded. Patients having device detected AHRE >5 min was taken as episodes of AT/AF.

### 2.5 Ethical justification and statistical analysis

The study protocol conforms to the ethical guidelines of the declaration of Helsinki and was reviewed and approved by the ethical committee of the institute (IEC no: INT/IEC/2019/002725).

All the statistical analysis was carried out using statistical package for social sciences (SPSS Inc., Chicago, IL, version 26.0 for windows). Variables are presented as mean ± SD or median (IQR). Variable were checked for outliers and normalcy (Shapiro–Wilk test). Differences between mean were analysed using independent paired *t*-test. Comparison between categorical variables was done using Chi-square test or Fisher exact test. A two-sided *p*-value <0.05 was considered to be significant for all variables. Subsequently, variables significantly associated with AHREs on univariate analysis were included in a multivariable regression analysis to identify independent predictors of AHREs.

## 3 Results

### 3.1 Patient characteristics

The study population comprised of 100 patients with 65% of all patients being males. The mean age of the study group was 56.2 ± 14.9 years. Hypertension was the commonest comorbidity present in 52% of the patients followed by diabetes (21%) and coronary artery disease (9%). The mean CHA_2_DS_2_VASc score of the study population was 1.8 ± 1.1 and 61% of all patients had a CHA_2_DS_2_VASc score ≥ 2.

Atrioventricular block (88%) was the commonest indication for PPI followed by sinus node dysfunction (12%). Majority of the patients had a good left ventricular systolic function as reflected by a mean ejection fraction of 52.5 ± 6.9%. The mean left atrial volume of the study group was 30.46 ± 4.5 mL and the mean left atrial volume index was 17.4 ± 2.9 mL/m^2^.

### 3.2 Device interrogation and lead parameters

AHRE detection feature with atrial high rate cut-off being 190 bpm was enabled in all the patients. Right atrial appendage was the site of right atrium lead implantation in all patients, while the ventricular leads were place at the septum in 64% and the remaining 36% patients had lead placed at the apex. PPI interrogation was performed at a mean duration of 15.2 ± 7.5 months after device implantation. Various lead parameters of the cohort analysed during the interrogation have been described in Table 1.

**Table 1:** Device and lead parameters during interrogation.

Lead parameter	Value
**Ventricular lead position**	
Apical	64%
Septal	36%
**Atrial lead parameters**	
Cumulative atrial pacing (%)	26.9 ± 29.8
Lead threshold (V)	0.73 ±0.52
Lead sensitivity (V)	2.94 ±1.77
Lead impedance (Ω)	515.4 ± 126.9
**Ventricular lead parameters**	
Cumulative ventricular pacing (%)	71.0 ± 39.7
Lead threshold (V)	0.86 ±0.44
Lead sensitivity (V)	10.54 ±3.87
Lead impedance (Ω)	590.2 ± 106.1

### 3.3 Incidence and clinical predictors of AHRE

Out of 100 participants with dual-chamber PPI, 17% patients had AHRE ≥5 minutes any time from one month after device implantation. Amongst these 17 patients who had AHRE, the majority of them (16 patients) had AHRE lasting <1 h and only 1 patient had an AHRE lasting >1 h.

Various demographic, clinical, echocardiographic and lead parameters were assessed to identify the predictors of AHRE amongst the population (Table 2). AHREs occurred more often in patients with sinus node disease (33.3%) than those with AV block (14.7%) as the indication for PPI. Only parameters that were significantly associated with AHRE on univariate analysis included the presence of diabetes (41.2% vs 16.9%; p = 0.04), apical position of the ventricular lead (64.7% vs 30.1%; p = 0.01), a lower atrial lead impedance (461.0 ± 69.6 vs 526.6 ± 133.2; p = 0.009) and a lower ventricular lead impedance (515.3 ± 132.9 vs 605.6 ± 93.6; p = 0.001). Further, regression analysis was performed to identify the parameters independently contributing to AHRE. Only apical lead position was found to have an independent association with AHREs (OR: 3.50, 95% CI: 1.02–12.03; p = 0.04) (Table 3).

**Table 2:** Predictors of Atrial high rate events on device interrogation.

Variable	AHREs (n = 17)	No AHREs (n = 83)	P- value
Age, years, mean (±SD)	59.3 (±17.5)	55.6 (±14.3)	0.35
Sex, n (%)			
Male	11 (64.7%)	52 (65.1%)	0.97
Female	6 (35.4%)	29 (34.9%)	
Diabetes, n (%)	7 (41.2%)	14 (16.9%)	**0.04**
Hypertension, n (%)	6 (35.3%)	46 (55.4%)	0.13
CAD, n (%)	0	9 (10.8%)	0.15
CHA_2_DS_2_-VASc score, mean ± (SD)	1.5 ± 1.2	1.9 ± 1.0	0.20
**Echocardiographic parameters**			
LVEF, %, mean ± (SD)	51.3 ± 7.6	53.1 ± 6.2	0.33
Left atrial volume (mL) mean ± (SD)	32.9 ± 5.6	29.9.3 ± 4.1	0.07
Indexed left atrial volume (mL/m^2^) mean ± (SD)	19.1 ± 4.7	17.1 ± 2.2	0.11
**Pharmacological therapy**			
Amiodarone, n (%)	2 (11.8%)	8 (9.6%)	0.26
Beta-blockers, n (%)	12 (70.6%)	55 (66.2%)	0.67
ACE inhibitors/ARBs, n (%)	6 (35.3%)	30 (36.1%)	0.78
Calcium channel blockers, n (%)	4 (23.5%)	15 (18.1%)	0.22
Antiplatelet agents, n (%)	12 (70.6%)	64 (77.1%)	0.45
Statins, n (%)	10 (58.8%)	56 (67.5%)	0.24
**Device and lead related parameters**			
Duration of implant (months) mean ± (SD)	16.1 ± 6.4	15.0 ± 7.7	0.09
Ventricular lead position (%):-			
Apical	11 (64.7%)	25 (30.1%)	**0.01**
Septal	6 (35.3%)	58 (69.9%)	
**Atrial lead parameters**			
Cumulative atrial pacing (%)	26.6 ± 30.2	27.0 ± 29.9	0.95
Lead threshold (V) mean ± (SD)	0.69 ± 0.28	0.75 ± 0.56	0.94
Lead sensitivity (V) mean ± (SD)	2.4 ± 1.6	3.1 ± 1.8	0.15
Lead impedance (*Ω*) mean ± (SD)	461.0 ± 69.6	526.6 ± 133.2	**0.009**
**Ventricular lead parameters**			
Cumulative ventricular pacing (%) mean ± (SD)	58.5 ± 47.5	73.5 ± 37.8	0.20
Lead threshold (V) mean ± (SD)	0.87 ± 0.35	0.86 ± 0.46	0.61
Lead sensitivity (V) mean ± (SD)	9.5 ± 2.9	10.7 ± 4.0	0.35
Lead impedance (*Ω*) mean ± (SD)	515.3 ± 132.9	605.6 ± 93.6	**0.001**

Abbreviations: AHREs: atrial high rate events; CAD: coronary artery disease; LVEF: left ventricular ejection fraction. ACE: angiotensin receptor convertase enzyme; ARBs: angiotensin receptor blocking agents.

**Table 3:** Binary logistic regression analysis.

Risk factors	Multivariable analysis		
	OR	CI (95%)	P-value
Diabetes	2.77	0.72–10.65	0.13
**Apical position of ventricular lead**	**3.50**	**1.02**–**12.03**	**0.04**
Atrial lead impedance	1.0	0.99–1.00	0.45
Ventricular lead impedance	0.99	0.99–1.00	0.06

The mean left atrial volume (mL) was greater in patients having AHREs although it did not reach statistical significance (32.88 ± 5.66 vs 29.9 ± 4.1; p = 0.07).

## 4 Discussion

There is limited data available on the incidence of AHREs amongst Indian population and its predictors. In our study, the incidence of AHREs was 17%, which is lower compared to the previous studies. The likely explanation for the same is that we excluded the patients with preimplantation AF and AF occurring within one month after device implantation in order to find the independent predictors of AHREs. In the study by Gillis et al, which included patients with preimplant AF, the incidence of AF after pacemaker implantation was 55%. Unlike our study, it also included patients within one month after implant and the mean time to occurrence of AF was 21 days which might also explain the lower incidence of AF in our study.^8^ Similarly, in the study by Healey et al, the incidence was 55.3%.^6^ However, the studies like Cheung et al and Wu et al which excluded patients with preimplant AF had an incidence of 29% and 26% respectively, which was comparable to the present study.^9^^,^^10^

It was also observed that in the current study, AF occurred more often in patients with sinus node disease (33.3%) than those with AV block (14.7%). This was similar to the observations made by Gillis et al which had an incidence of 68% among sinus node disease and 37% among AV block.^8^ Among patients with AHREs in our study, 94.1% had an AHRE for a duration of <1 h and only 5.9% had for >1 h which is contrary to other studies like Cheung et al, where the 39% having AHRE <1 h and 61% having AF > 1 h.^9^ Again, the likely explanation was the inclusion of patients with preimplant AF, which with time are known to have increased burden of AF.

Chen et al showed that in sick sinus syndrome patients, atrial pacing ≥60% is an independent risk factor for AF.^11^ However, Cheung et al and Wu et all showed that higher ventricular pacing was associated with AF in SSS.^9^^,^^10^ Such associations were not found in our study (Table 4).

**Table 4:** Various studies comparing the incidence of AHREs.

Study	Number of patients	Incidence of AHREs	Remarks
Gillis et al, 2002	231	−55%	- Included patients with preimplant AF and within one month after pacemaker implantation
Stambler et al, PASE trial, 2002	407	18%	- Patients with preimplant chronic AF were excluded
			- Included both single and dual-chamber pacing
Lama et al, 2002	2010	24.2%	- Included patients with preimplant AF
			- Included both single and dual-chamber pacing
Cheung et al, 2006	262	29%	- Excluded patients with preimplant AF
Healey et al, 2013	445	55.3%	- Included patients with preimplant AF
Wu et al, 2020	219	26%	- Excluded patients with preimplant AF
Our study	100	17%	- Only Dual-chamber pacemaker patients
			- Excluded patients with preimplant AF

Abbreviations: AHREs: atrial high rate events; AF: atrial fibrillation.

Another significant finding from our study includes an independent relationship of AHREs with right ventricular (RV) apical pacing compared to septal pacing. Evidence points to the deleterious effects of apical pacing on cardiac hemodynamics. RV apical pacing causes electrical activity to propagate from myocardium rather than His – Purkinje conduction system, producing a left ventricular electrical activation sequence similar to left bundle-branch block and with a longer QRS duration than RV septal pacing. This can lead to LV contractions which are less efficient than that with RV septal pacing causing more desynchronization and resultant increased end diastolic ventricular and atrial pressures.^12^ On the contrary, RV septal pacing initiates ventricular depolarization in the septal wall across the mitral septal papillary muscle, where pacing activation starts and more closely resembles the normal pattern of electrical activation resulting in a narrower QRS and lower end diastolic pressures.^13^

## 5 Limitations

1) We cannot tell with certainty that all patients included in the study were free from AF before PPI as some may have had silent AF beforehand.
2) Since all the patients found to have AHRE were only detected on device interrogation and not on electrocardiogram recordings, it can be difficult to differentiate with certainty AF from other forms of atrial tachyarrhythmias.
3) Since this was a single time, cross-sectional, observational study, the pacemaker settings changes and other clinical parameters couldn't be compared at various time points between pacemaker implantation and occurrence of AF
4) Atrial undersensing can occur during AHRE episodes and can result in not detecting the AHREs.
5) The patients with AHRE >1 h were very few in our study and hence the clinical predictors of AF in these patients couldn't be commented.
6) The clinical outcome of patients who developed AF was not known and merits further research in that area

## 6 Conclusion

This is the first study conducted in Indian population on AF occurrence after dual-chamber PPI. Our study found that in Indian patients, the incidence of AF after one month of PPI was 17% which was comparable to other western studies. Right ventricular apical pacing was independently associated with AHREs in our study.

## Declarations

### Ethics approval and consent to participate

The study protocol conforms to the ethical guidelines of the declaration of Helsinki and was reviewed and approved by the ethical committee of the institute (IEC no: INT/IEC/2019/002725) (Reference no: NK/5778/MD/665 dated 26th December 2019).

### Consent for publication

Written informed consent was obtained directly from the patients for publication original research article.

### Availability of data and materials

All data and materials will be upload as per the needs of the editor/reviewer or the readers as per their request.

### Competing interests

None.

### Funding

None.

### Authors’ contributions

Patient management- JS, AB, PP, YPS, PB; Writing the manuscript- JS, AB, PP, YPS, PB, NK, SYS, BS, PCR; Reviewing- PP, YPS, PB; Key insights- PB, PP, YPS; Revision- AB, PP; Approval and supervision- YPS.

### Acknowledgements

None.
